# The Hand, Foot, and Mouth Disease Sentinel Surveillance System in South Korea: Retrospective Evaluation Study

**DOI:** 10.2196/59446

**Published:** 2024-07-23

**Authors:** Bryan Inho Kim, Chiara Achangwa, Seonghui Cho, Jisoo Ahn, Jisu Won, Hyunkyung Do, Dayeong Lee, Bohye Yoon, Joohee Kim, Sukhyun Ryu

**Affiliations:** 1 Division of Infectious Disease Control, Korea Disease Control and Prevention Agency, Cheongju-si, Republic of Korea; 2 Department of Preventive Medicine, College of Medicine, The Catholic University of Korea, R6117, Omibus Park, 222 Banpo-daero, Seocho-gu, Seoul, 06591, 82 0231478383, 82 025323820, Republic of Korea; 3 Department of Preventive Medicine, Konyang University College of Medicine, Daejeon, Republic of Korea

**Keywords:** hand-foot-mouth disease, surveillance, evaluation, sentinel, sensitivity, surveillance system, effective, South Korea, COVID-19, public health system, policy maker, timeliness, stability, completeness, representativeness, hand, foot, and mouth disease

## Abstract

**Background:**

South Korea has implemented a hand, foot, and mouth disease (HFMD) surveillance system since 2009 to monitor incidence trends and identify disease burden. This nationwide surveillance involves a network of approximately 100 pediatric clinics that report all probable and confirmed HFMD cases. Following the COVID-19 pandemic, infectious disease surveillance systems must be evaluated to ensure the effective use of limited public health resources.

**Objective:**

This study aimed to evaluate the HFMD sentinel surveillance system in South Korea from 2017 to 2022, focusing on the transition period after the COVID-19 pandemic.

**Methods:**

We retrospectively reviewed the HFMD sentinel surveillance system from the Korea Disease Control and Prevention Agency using systematic guidelines for public health surveillance system evaluation developed by the US Centers for Disease Control and Prevention. We assessed the system’s overall performance in 5 main factors: timeliness, stability, completeness, sensitivity, and representativeness (ie, the age and geographic distribution of sentinels). We rated these factors as weak, moderate, or good.

**Results:**

Our study showed that the completeness, sensitivity, and age representativeness of the HFMD surveillance performance were temporarily reduced to moderate levels from 2020 to 2021 and recovered in 2022, while the timeliness and geographic representativeness were maintained at a good level throughout the study period. The stability of the surveillance was moderate from 2017 to 2021 and weak in 2022.

**Conclusions:**

This is the first study to evaluate the HFMD surveillance system after the acute phase of the COVID-19 pandemic. We identified a temporarily reduced level of performance (ie, completeness, sensitivity, and age-specific representativeness) during the acute phase of the pandemic and good performance in 2022. Surveillance system evaluation and maintenance during public health emergencies will provide robust and reliable data to support public health policy development. Regular staff training programs and reducing staff turnover will improve HFMD surveillance system stability.

## Introduction

Hand, foot, and mouth disease (HFMD) is a highly transmissible pediatric infectious disease characterized by a rash or vesicular appearance on the hands, feet, and tongue [1]. The clinical symptoms of HFMD are mostly mild and usually self-limiting. However, HFMD was estimated to cause 97,000 disability-adjusted life-year losses per annum across Asia [2]. Furthermore, neurologic complications are associated with increased mortality, especially in children [3]. In South Korea, HFMD epidemics recur between April and August, and the annual economic burden is estimated to be US $100 million [4,5]. Seasonal HFMD epidemics have been a major public health concern in South Korea.

South Korea has had an HFMD surveillance system since 2009 to monitor incidence trends and identify the HFMD burden; it is provided by the Korea Disease Control and Prevention Agency (KDCA) [6]. This nationwide surveillance system has been carried out through a network of approximately 100 pediatric clinics, which report the number of clinically diagnosed cases and the total number of outpatients weekly to the KDCA through web-based reporting systems [7]. These sentinel sites are designated based on the provincial-level population, and they monitor community-level HFMD trends across the nation. The data acquired from the HFMD sentinel sites are collected and analyzed weekly and disseminated through weekly reports on the KDCA website (Multimedia Appendices 1 and 2) [8]. The sentinel surveillance system provides information on community-level HFMD trends to support decision-making for effective public health measures. Surveillance system evaluation is emphasized by the US Centers for Disease Control and Prevention (CDC) in guidelines initially published in 1988 [9] and updated in 2001 [10].

It is important to evaluate the HFMD surveillance system as it provides data for key epidemiological findings, including seasonality and economic burden [2,4]. Previous studies report a decline in HFMD notifications during the acute phase of the COVID-19 pandemic [11,12]. However, as the majority of public health resources were focused on COVID-19 responses during the pandemic [13], the HFMD surveillance system must be evaluated to identify areas for improvement in a resource-limited public health sector [14]. However, no studies have evaluated HFMD surveillance during the transition period after the COVID-19 pandemic. This study aimed to review and systematically evaluate the HFMD surveillance system in South Korea from 2017 to 2022 based on the CDC surveillance evaluation guidelines.

## Methods

### Data Collection

We obtained the weekly number of HFMD notifications from outpatient clinics from the Korean HFMD surveillance system from January 1, 2017, to December 31, 2022 [8]. The data included the number of weekly reports and number of weeks published in the surveillance report. Furthermore, we reviewed the electronic records for the weekly number of notifications from the sentinel sites and surveillance operations to identify changes in the number of KDCA surveillance officers. Furthermore, we collected the monthly number of patients with HFMD from the Korean Health Insurance Review and Assessment Service (KHIRA) [15] from January 1, 2017, to December 31, 2022. The data from the KHIRA were based on the national health insurance reimbursement data of more than 80,000 health care providers in South Korea, covering around 97% of the South Korean population (46 million). It includes patient diagnoses using the *International Classification of Diseases, Clinical Modification, 10th Revision* (*ICD-10-CM*) [16].

### Ethical Considerations

The data were collected from KDCA’s web-based reporting system and include no personally identifiable information. Ethical approval was waived by the Institutional Review Board of Konyang University (KYU 2023-10-030).

### Evaluation Framework

We evaluated a nationwide HFMD surveillance system using a structured framework based on the updated CDC guidelines [10]. The evaluation attributes included timeliness, stability, completeness, sensitivity, and representativeness (Multimedia Appendix 3). These attributes have been widely used to evaluate infectious disease surveillance performance at the country level [17,18]. For consistency and comparability, we used a quantitative scoring scale from 1 to 3 for each indicator: weak (1), moderate (2), and good (3), from January 1, 2017, to December 31, 2022.

### Timeliness

For timeliness, we measured the mean time lag for each year during the study period between the HFMD case notification (from the physician at the sentinels to the KDCA), acquired from case-report records, and dissemination (from the KDCA to the public), collected from the HFMD surveillance reports (Multimedia Appendix 4). As HFMD sentinel sites report weekly, all data were formatted in weeks. We calculated the mean and SD of the time lag of the surveillance system’s weekly notifications each year. Statistically, this is expressed by the following equation:


$$\overset{-}{x}=\frac{\sum x}{n}$$

where *x* is each time lag between the case notification and results dissemination and *n* is the number of weeks in that year (ie, 52 wk). Furthermore, we provided the SD by using the following equation:


$$SD=\sqrt{\frac{\sum {\left(x-\;\overset{-}{x}\right)}^{2}}{n-1}}$$

We considered the timeliness of the surveillance performance as weak (score 1), moderate (score 2), and good (score 3) using a 3-point scale (score 1: a mean time lag ≥5 wk; score 2: a mean time lag of 3‐4 wk; and score 3: a mean time lag of ≤2 wk).

### Stability

We defined stability as how stable the surveillance system operates without interruption [9]. In this evaluation, we divided the monitoring system into 3 detailed categories: frequency of program suspension, staff replacement, and KDCA personnel training. We reviewed the surveillance operation records to identify the number of times that the surveillance system was halted for technical issues, including the blackout of the HFMD surveillance database system at the KDCA (score 1: >10 times; score 2: 6‐10 times; and score 3: <6 times). The web-based surveillance system was automatically programmed to record any technical issues and changes in the surveillance officers in charge. We reviewed the KDCA surveillance officer turnover each year (score 1: >300%; score 2: 100‐299%, and score 3:<100%). This is expressed by the following equation:


$$Turnover\;rate\;\left(\%\right)=\;\;\frac{Number\;of\;surveillance\;officers\;who\;left}{Average\;number\;of\;officers}\;\times 100$$

We also reviewed the number of times that the KDCA officers received surveillance-related training per year (score 1: none; score 2: 1‐2 times; and score 3: ≥3 times). These data were acquired from the KDCA web-based reporting and surveillance system’s electronic records. We calculated the mean of these 3 indicators each year to identify the overall stability (mean score between 1 and <2: weak; mean score of between 2 and <3: moderate; and mean score of 3: good).

### Completeness

In our assessment of completeness, we calculated the submission rate of complete notification reports out of the overall number of notification reports submitted by the sentinels to the KDCA each year. This is expressed by the following equation:


$$Completeness\;\left(\%\right)=\;(1-\;\frac{Number\;of\;observed\;notification\;reports\;per\;year}{Overall\;number\;of\;expected\;notification\;reports\;per\;year})\;\times 100$$

This value is automatically calculated in the web-based system. No reports from any sentinel site were considered missing reports. We considered the completeness of the surveillance performance as weak (score 1), moderate (score 2), and good (score 3) using a 3-point scale (score 1: <80%; score 2: 80‐90%; and score 3: >90%).

### Sensitivity

To assess sensitivity for the incidence trends, we obtained the monthly number of reported HFMD cases in the surveillance system from the KDCA [8]. Due to the limited public health resources for identifying the nationwide incidence of HFMD, we assumed that the number of patients from the KHIRA represents the HFMD incidence in the community. We collected the monthly number of patients clinically diagnosed with HFMD (*ICD-10-CM*: B08.4) from January 1, 2017, to December 31, 2022 [15]. Then, we calculated the correlation coefficient for each year between the monthly number of notified HFMD cases in the surveillance system and the number of new patients with HFMD from the KHIRA. We first conducted a Shapiro-Wilk test to determine if the variables were normally distributed. Then, we conducted either the Pearson or Spearman test, where appropriate. The Pearson test is expressed by the following equation:


$$\rho_{x,\;\;y}=\frac{cov\;\left(x,y\right)}{\sigma_{x}\sigma_{y}}$$

where ρ is the Pearson correlation coefficient between *x* (ie, the monthly number of notified HFMD cases from the KDCA) and *y* (ie, the monthly number of patients with HFMD from the KHIRA), cov is the covariance, $\sigma_{x}$ is the SD of *x*, and $\sigma_{y}$ is the SD of *y*. The Spearman test is defined as the Pearson test between the rank variables. We considered the sensitivity of the surveillance performance as weak (score 1), moderate (score 2), and good (score 3) using a 3-point scale (score 1: <0.7; score 2: 0.7‐0.79; and score 3: ≥0.8).

### Representativeness

For representativeness (ie, age representativeness), we compared the proportion of 0‐ to 6-year-olds in the surveillance data, which is the age group most affected by HFMD [19], with the proportion of 0‐ to 6-year-olds from KHIRA data on patients with HFMD. We considered the representativeness of the surveillance performance as good (score 3) if the difference in the proportion was less than 10%, moderate (score 2) if the difference was from 10% to 20%, and weak (score 1) if the difference was more than 20%. Furthermore, we assessed the geographic representativeness of the sentinel surveillance sites by comparing the geographical distribution of the sentinel sites with the population distribution obtained from the Korean Statistical Information Service [20]. Using the overall designated number of sentinel sites in South Korea, we calculated the expected number of sentinel sites in 17 regions, including 8 metropolitan cities and 9 provinces, by the regional population. This is expressed by the following equation:


$$Expected\;number\;of\;sentinel\;sites=\;\frac{Overall\;number\;of\;sentinel\;sites\;\times \;Population\;in\;the\;region\;}{Total\;population\;in\;South\;Korea}$$

We then conducted a chi-square test to determine the statistical difference between the expected number of sentinels and the observed number of sentinels in the region. This can be expressed by the following equation:


$$X^{2}=\;\sum_{i=1}^{k}\frac{{\left(O_{i}-E_{i}\right)}^{2}}{E_{i}}$$

where *O_i_* is the observed number of sentinels in each region, *E_i_* is the expected number of sentinels in each region, and *k* is the number of regions. We considered the geographic representativeness of the surveillance performance to be good (score 3) if the *P* value of the test is above .05 and weak (score 1) if the *P* value of the test is less than .05. We conducted all analyses using RStudio (version 4.1.0; Posit).

## Results

**Figure 1.**
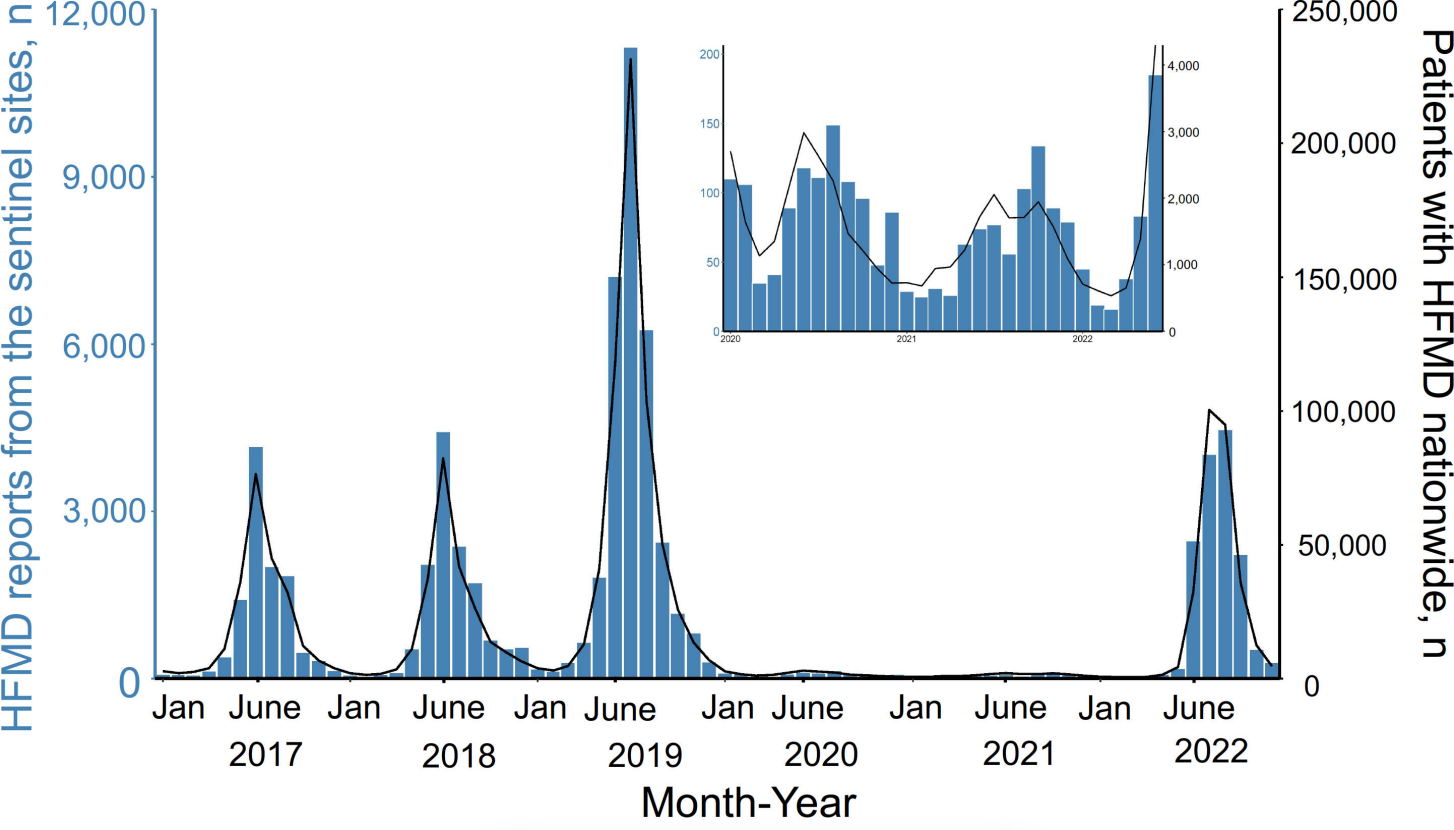
Monthly number of the hand, foot, and mouth disease (HFMD) sentinel surveillance system notifications from outpatient clinics and the monthly number of patients with HFMD from a nationwide reimbursement system (Korean Health Insurance Review and Assessment Service) in South Korea, from 2017 to 2022. The blue vertical bar indicates the number of HFMD notifications and the black solid line indicates the number of patients with HFMD in South Korea.

The mean number of HFMD sentinel sites between 2017 and 2022 was 102 (Table 1). The weekly number of HFMD reports increased from April to August, peaking in late June or July, during the period between 2017 and 2019 and in 2022. We could not identify this seasonal pattern (fewer than 150 cases per month; Figure 1) in the period between 2020 and 2021.

**Table 1.** The timeliness, stability, data completeness, and sensitivity of the hand, foot, and mouth disease (HFMD) surveillance system in South Korea, from 2017 to 2022.

Factors		Year					
		2017	2018	2019	2020	2021	2022
Sentinel sites, n		93	97	97	109	109	110
**Timeliness**							
	Time lag from the report to the dissemination of the collected data (week), mean (SD)	1.80 (1.40)	1.69 (1.57)	1.23 (0.43)	1.01 (0.14)	1.01 (0.01)	1.01 (0.14)
**Stability**							
	Suspensions for the surveillance system, n	0	0	0	0	0	0
	Turnover rate^a^ for the KDCA^b^ surveillance officers	N/A^c^	N/A	N/A	N/A	N/A	260%
	Training for the KDCA officers, n	0	0	0	0	0	0
**Completeness**							
	Reporting rate^d^	0.99	0.94	0.95	0.88	0.87	0.93
**Sensitivity**							
	Correlation coefficient^e^	0.99	0.99	0.99	0.70	0.77	0.97
	*P* value	<.01	<.01	<.01	.01	<.01	<.01

a $Turnover\;rate\;\left(\%\right)=\;\frac{Number\;of\;surveillance\;officers\;who\;left}{Average\;number\;of\;officers}\;\times 100$.

b KDCA: Korea Disease Control and Prevention Agency.

c N/A: not available.

d Reporting rate is defined as the observed number of reports compared to the expected number of reports in a year (ie, 52 wk).

e Correlation coefficient: the coefficient between the number of monthly HFMD case notifications from the KDCA and the monthly number of patients with HFMD from the Korean Health Insurance Review and Assessment Service was obtained by the Pearson or Spearman test, where appropriate.

### Timeliness

In the timeliness assessment, we found a shorter than 1.9-week lag between the case notification and data dissemination during the study period. This was deemed good (score 3) based on our criteria.

### Stability

For stability, we could not identify any HFMD surveillance system cessation during the study period (score 3, good performance). However, there was no regular surveillance training program for surveillance staff at the KDCA (score 1, weak performance). Due to data limitations, we only assessed the turnover rate of the KDCA surveillance staff as 260% during 2022 (score 1, weak performance). Therefore, the mean stability was moderate (mean score of 2) from 2017 to 2021 and weak (mean score of 1.6) in 2022 (Table 1).

### Completeness

Regarding data completeness, the complete report rate, including zero case reports, was above 90% (good performance) from 2017 to 2019 and in 2022. It was 88.1% and 88.6% (moderate performance) in 2020 and 2021, respectively (Table 1).

### Sensitivity

For the sensitivity, the correlation coefficient between the number of monthly HFMD case notifications from the sentinel sites and the monthly number of patients with HFMD from KHIRA was >0.97 (good performance) from 2017 to 2019 and in 2022, while it was 0.70 and 0.77 (moderate performance) in 2020 and 2021, respectively (Table 1 and Multimedia Appendix 5).

### Representativeness

We identified that the difference between the proportions of 0‐ to 6-year-olds from the HFMD surveillance system by the KDCA and among the patients with HFMD from the KHIRA ranged from 3% to 6% (score 3, good performance) from 2017 to 2019 and in 2022 and ranged from 12% to 13% (score 2, moderate performance) from 2021 to 2022 (Table 2). In our assessment of the representativeness of the geographic distributions of the sentinel sites, the *P* values from chi-square tests were between .95 and .99 from 2017 to 2022 (good performance) (Table 3).

**Table 2.** The age representativeness of the hand, foot, and mouth disease (HFMD) surveillance system in South Korea, from 2017 to 2022. We collected data for the number of HFMD notifications from the Korea Disease Control and Prevention Agency (KDCA) and the number of patients with HFMD from the Korea National Health Insurance and Review Assessment Service (KHIRA).

Data source and variable		Year					
		2017	2018	2019	2020	2021	2022
**Sentinel surveillance data from the KDCA**							
	<7-years-olds, n	4612	5599	10,477	452	434	11,817
	Overall HFMD reports, n	4833	5933	11,049	487	478	12,326
	Proportion of <7-year-olds (%)	95.4	94.4	94.8	92.8	90.8	95.9
**Health insurance reimbursement data from the KHIRA**							
	<7-years-old, n	221,489	219,814	566,762	19,360	14,597	275,124
	Overall patients with HFMD, n	241,871	242,494	637,131	22,974	18,431	300,635
	Proportion of <7-years-old (%)	91.6	91	89	79.9	79.2	91.5
Differences between the proportions from the KDCA and KHIRA (%)		3.8	3.4	5.8	12.9	11.6	4.4

**Table 3.** The geographic representativeness of the hand, foot, and mouth disease (HFMD) surveillance system in South Korea, from 2017 to 2022. We obtained the designated number of sentinel sites from the Korea Disease Control and Prevention Agency (KDCA). We used the overall number of designated sites in each year to calculate the expected number of sentinel sites in 17 regions, including 8 metropolitan cities and 9 provinces, by the regional population. E: Expected; D: Designated.

Regions	Year and sentinel sites, n											
	2017		2018		2019		2020		2021		2022	
	E^a^	D	E	D	E	D	E	D	E	D	E	D
Overall	—^b^	93	—	96	—	99	—	114	—	112	—	110
Seoul	15	17	16	16	16	17	18	17	18	16	17	16
Pusan	5	6	6	6	7	7	7	6	6	6	6	6
Daegu	4	5	5	5	5	5	5	6	5	6	5	6
Incheon	5	5	6	6	6	6	7	8	6	8	6	8
Gwangju	3	3	3	3	3	3	4	3	4	3	3	2
Daejeon	3	2	3	3	3	3	3	3	3	3	3	3
Ulsan	2	1	2	1	2	3	3	3	3	3	3	3
Sejong	1	1	1	1	1	1	1	1	1	1	1	1
Gyeonggi	26	23	26	23	28	23	32	27	32	26	32	26
Kangwon	3	3	3	3	3	3	3	6	3	6	3	6
Chungbuk	3	3	3	3	3	3	4	3	3	3	3	3
Chungnam	4	3	4	3	4	3	5	3	5	3	5	3
Jeonbuk	3	4	3	4	3	4	4	5	4	5	4	5
Jeonnam	3	4	3	4	3	4	4	5	4	5	4	5
Kyungbuk	4	5	5	5	5	5	5	6	5	7	5	7
Kyungnam	6	6	7	7	7	7	8	8	8	8	7	7
Jeju	1	2	1	2	1	3	2	3	2	3	2	3
*χ*^2^ test(*df*)	3.93(16)	—	2.78(16)	—	6.37(16)	—	6.91(16)	—	8.06(16)	—	7.90(16)	—
*P* value	.99	—	.99	—	.98	—	.98	—	.95	—	.95	—

a $Expected\;number\;of\;sentinel\;sites=\frac{Overall\;number\;sentinel\;sites\;\times \;Population\;in\;the\;region\;assessed}{Total\;population\;in\;South\;Korea}$.

b Not applicable.

## Discussion

This is the first study to evaluate the HFMD surveillance system in South Korea. Our study findings indicate that the completeness, sensitivity, and age-specific representativeness of HFMD surveillance in South Korea decreased to moderate levels from 2020 to 2021 but recovered in 2022.

### Timeliness

Timeliness is an important measure to evaluate in any surveillance system. Case notification is the result of a chain of events from infection to report dissemination by public health authorities [21]. Delays in the processes originate from patient delay (ie, delays in visiting the clinics), doctor delay (ie, time delay from consultation to diagnosis), laboratory delay (ie, time delay from the laboratory test to confirmed test result), and notification delay (ie, time delay from the physician to the appropriate public health authority) [22]. The Korean HFMD surveillance system collects the information regarding the notification date and dissemination of the case report (Multimedia Appendix 4). Therefore, in this study, we only accounted for the time lag from the case notification to results dissemination (ie, time elapsed between the notification from the physician to the dissemination of the surveillance report). As HFMD reporting involves a web-based reporting system in the KDCA, there was a shorter than 2-week time lag between case notification from the sentinel clinics and weekly surveillance report dissemination. A previous study demonstrated that quantifying the reporting delay is necessary to manage outbreaks and communicate with the public [21,22]. Therefore, this finding provides valuable input to develop real-time epidemic models using surveillance data.

As surveillance system timeliness differs by country and disease [23], additional study of HFMD surveillance systems in other countries is necessary to compare results. The Korean HFMD surveillance system depends on sentinel participation, and there is no penalty for delayed reporting. An electronic reporting system is now standardized in many countries, and it improves the timeliness and completeness of infectious disease surveillance [24]. Additional evaluation of timeliness is warranted to identify the specific time lag in each reporting step [23].

### Stability

We identified that the operational stability of the surveillance system was good. However, due to the replacement of surveillance officers and lack of KDCA training programs, it was evaluated as moderate from 2017 to 2021. The more frequently infectious disease surveillance personnel are replaced, the more likely it is that the surveillance system stability will be affected. It may become difficult to derive improvement measures because the relevant indicators cannot be calculated. Therefore, personnel turnover must be minimized and an adequate staffing level secured to manage surveillance system stability.

KDCA surveillance officers conduct routine public health activities. Many countries, including South Korea, have initiated programs to train field epidemiologists in public health surveillance and outbreak responses [25]. However, we identified no regular training program for surveillance staff in South Korea. Many countries struggle to attract and retain public health personnel in the field of infectious diseases due to the high level of responsibility and the principle of learning by doing [26,27]. Furthermore, limited academic resources, including a population-based training program and a lack of proper teaching methodologies, result in insufficient opportunities for professional development [27]. Therefore, to improve stability, tying surveillance officers to the academic field will support professional development and sustainability.

### Completeness

The notification data quality is important to enable public health authorities to respond in a timely and appropriate manner. Therefore, it is necessary to complete all data fields in the notification system [24,28]. The current Korean HFMD electronic surveillance system is mainly operated by web-based reporting records to track reporting at each sentinel site. The system identifies missing fields on the notification form, and an alarm is sent to the sentinel sites to complete the missing data. In this study, we could not identify any missing data fields in the reporting system. Thus, we considered the completeness of data as good during the study period. However, we identified that the completeness of notification was decreased from 2020 to 2021 and recovered in 2022. This is likely due to surges in the number of COVID-19 notifications.

### Sensitivity

Sensitivity evaluation using community-based investigation requires additional resources, and it is difficult to conduct regularly. Previous studies demonstrated alternative methods for evaluating sensitivity by comparing other sources of surveillance data, including public health laboratories, hospitals, and the national reimbursement system [14,29,30]. Previous studies report that the sensitivities of HFMD surveillance were from 38% to 57% and 76% in Hong Kong and Thailand, respectively [14,29]. However, the study design, including data sources, population coverage, and sensitivity measuring methods, differs between studies, warranting caution when comparing the results. A surveillance system with low sensitivity can be useful for monitoring disease outbreak trends if sensitivity remains constant over time [10]. In our study, to overcome the limitation of alternative methodologies and identify sensitivity changes over time, we evaluated 6 consecutive years (2017‐2022) of data. We identified lower sensitivity during the 2020‐2021 period compared to other years. This is likely due to the significantly reduced number of HFMD cases due to different public health and social distancing measures in different locations (ie, different measures in metropolitan areas and provincial regions). Additional study is necessary to determine the association between surveillance sensitivity and behavior changes, such as visiting clinics to treat HFMD. As HFMD is normally not a life-threatening disease, patient visits to clinics may be affected by the overall number of COVID-19 cases in the country.

### Representativeness

The nationwide reimbursement data have been widely used to identify the incidence of diseases in South Korea [5,16,31]. To determine age-specific representativeness of the surveillance system, we compared the age-specific proportion of the incidence data between the KDCA and KHIRA. From the national health insurance reimbursement data, we identified that the proportion of 0‐ to 9-year-olds accounted for more than 91% to 98% of HFMD incidence in South Korea during the study period. Children aged 0‐6 years old accounted for 89% to 92% of the HFMD incidence from 2017 to 2019 and in 2022 and 79% to 80% from 2020 to 2021. The lower HFMD incidence in the 0‐6 years age group from 2020 to 2021 compared to other years is likely due to the strict public health measures in place during the COVID-19 pandemic from 2020 to 2021, including closing kindergartens and wearing face masks. This result is in line with a previous Korean study that determined that public health and social measures are strongly associated with reduced HFMD transmissibility, particularly in kindergarteners [5].

It is important to select sentinel locations to effectively meet surveillance objectives and save public health resources. We identified that the geological distribution of HFMD sentinels in South Korea was good during the study period. However, having more sentinels in underrepresented areas will enhance the local resolution of HFMD occurrence. Even if the current sentinel sites are based on population, other factors, including location, distance between sites, and population density, should be considered when designating new sentinel sites [32].

### Limitations

There are several limitations to this study. First, we could not evaluate other surveillance performance attributes, including simplicity, flexibility, and acceptability. An additional survey using a questionnaire is warranted to evaluate these other attributes. Second, we could not evaluate the positive predictive value. As HFMD surveillance is based on the reporting of clinical symptoms, further study is necessary to identify its positive predictive value (ie, the proportion of cases with confirmed pathogens out of the number of reported cases).

### Conclusions

In conclusion, the completeness, sensitivity, and age representativeness of the HFMD surveillance system were temporarily reduced during the acute period of the COVID-19 pandemic. Continuous evaluation of surveillance performance will provide robust and reliable data to support public health policy development.

## Supplementary material

10.2196/59446 Multimedia Appendix 1 Flow of the hand, foot, and mouth disease notification and result report release in the surveillance system in South Korea.

10.2196/59446 Multimedia Appendix 2 Notification form of hand, foot, and mouth disease in South Korea.

10.2196/59446 Multimedia Appendix 3 Definitions of the surveillance performance factors based on guidelines from the US Centers for Disease Control and Prevention: timeliness, stability, completeness, sensitivity, and representativeness.

10.2196/59446 Multimedia Appendix 4 Time elapsed from infection to dissemination of the notification result to the public in the hand, foot, and mouth disease surveillance system in South Korea.

10.2196/59446 Multimedia Appendix 5 The correlation coefficient between the number of monthly hand, foot, and mouth disease (HFMD) case notifications from the Korea Disease Control and Prevention Agency (KDCA) and the monthly number of patients with HFMD from the Korea Health Insurance Review and Assessment Service (KHIRA).

## Abbreviations

CDC: Centers for Disease Control and Prevention
HFMD: hand, foot, and mouth disease
*ICD-10-CM*: *International Classification of Diseases, Clinical Modification, 10th Revision*
KDCA: Korea Disease Control and Prevention Agency
KHIRA: Korean Health Insurance Review and Assessment Service
