# Supplementary material for: The Hand, Foot, and Mouth Disease Sentinel Surveillance System in South Korea: Retrospective Evaluation Study
Source: JMIR Public Health Surveill. 2024 Jul 23;10:e59446. doi: 10.2196/59446 (PMC11287233; doi:10.2196/59446)
Supplement: Multimedia Appendix 2 [file publichealth-v10-e59446-s002.docx]

**Appendix 2.** Notification form of hand, foot, and mouth disease in South Korea.

| **HFMD case notification form**  **Receipt:** Director of the Korean Diseases Control and Prevention Agency (KDCA)  Period of surveillance: Week (YYYY/MM/DD – YYYY/MM/DD)   \| Age-Classification \| 0 year \| 1-6 years \| 7-12 years \| 13-18 years \| \| --- \| --- \| --- \| --- \| --- \| \| Number of patients visited \|  \|  \|  \|  \| \| Number of patients diagnosed with hand foot and mouth disease \|  \|  \|  \|  \|   Notification date: YYYY/MM/DD  Head of the sample monitoring agency:  Name of the sentinel:  Institution code: Phone number: |
| --- | --- | --- | --- | --- | --- | --- | --- | --- | --- | --- | --- | --- | --- | --- | --- |
