# Supplementary material for: The Hand, Foot, and Mouth Disease Sentinel Surveillance System in South Korea: Retrospective Evaluation Study
Source: JMIR Public Health Surveill. 2024 Jul 23;10:e59446. doi: 10.2196/59446 (PMC11287233; doi:10.2196/59446)
Supplement: Multimedia Appendix 3 [file publichealth-v10-e59446-s003.docx]

**Appendix** **3.** Definitions of the surveillance performance factors based on guidelines from the U.S. Centers for Disease Control and Prevention: timeliness, stability, completeness, sensitivity, and representativeness

| Attributes | Definition |
| --- | --- |
| Timeliness | Timeliness is the time required for reporting/analysis/dissemination within the surveillance system. |
| Stability | Stability is the reliability (ability to properly collect, manage, and provide data without error) and availability (ability to operate when necessary) of the surveillance system. |
| Completeness | Completeness is how completely the data to be collected through the surveillance system is reported. |
| Sensitivity | Sensitivity is the proportion of cases reported through the surveillance system among the total cases in the community. |
| Representativeness | Representativeness refers to how accurately the system describes the distribution of disease occurrence across people and places in a population over time. |
