# Supplementary material for: The Hand, Foot, and Mouth Disease Sentinel Surveillance System in South Korea: Retrospective Evaluation Study
Source: JMIR Public Health Surveill. 2024 Jul 23;10:e59446. doi: 10.2196/59446 (PMC11287233; doi:10.2196/59446)
Supplement: Multimedia Appendix 4 [file publichealth-v10-e59446-s004.docx]

**Appendix 4.** Time elapsed from infection to dissemination of the notification result to the public in the hand, foot, and mouth disease surveillance system in South Korea.


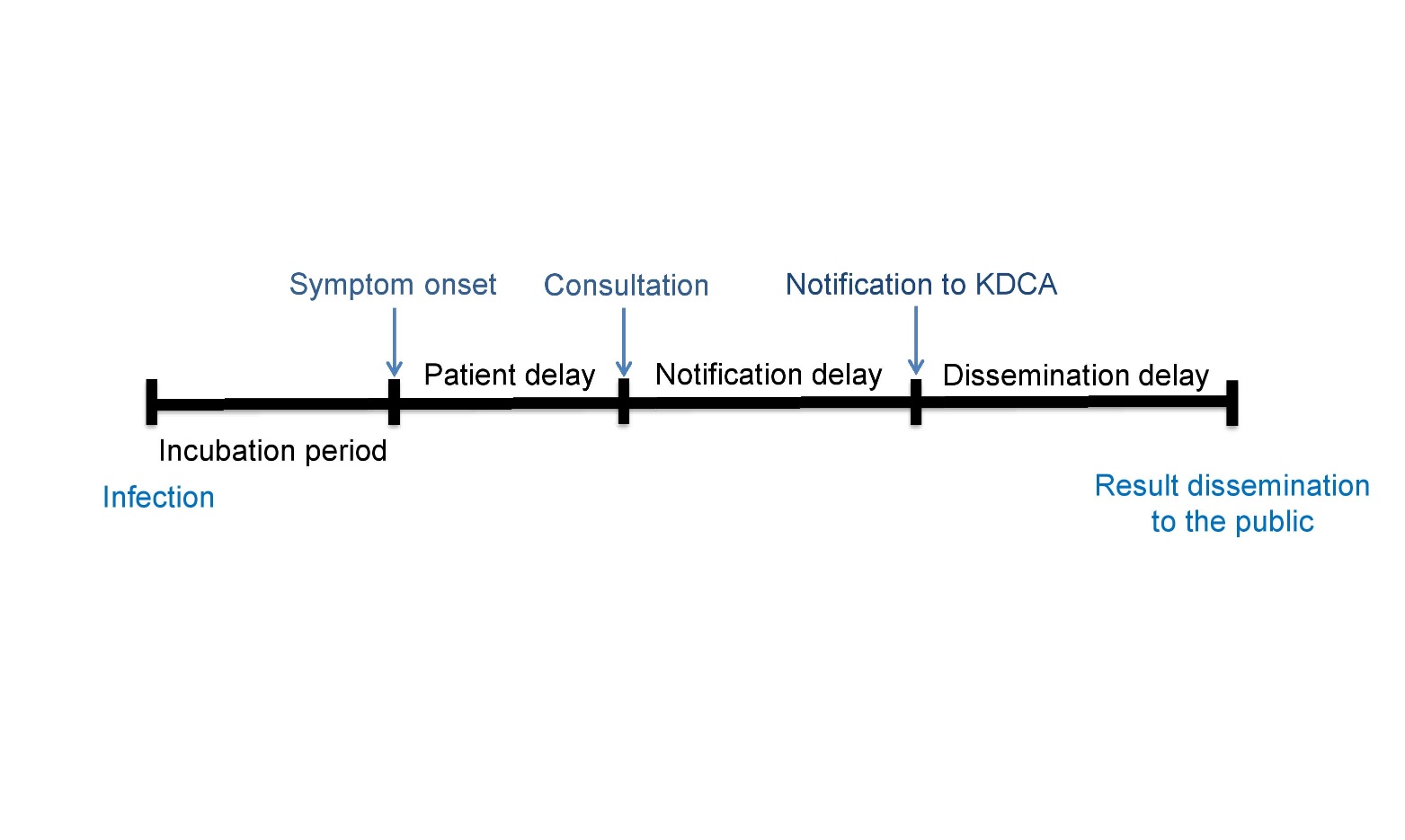


Note. KDCA: Korea Disease Control and Prevention Agency
