# Supplementary material for: The Hand, Foot, and Mouth Disease Sentinel Surveillance System in South Korea: Retrospective Evaluation Study
Source: JMIR Public Health Surveill. 2024 Jul 23;10:e59446. doi: 10.2196/59446 (PMC11287233; doi:10.2196/59446)
Supplement: Multimedia Appendix 5 [file publichealth-v10-e59446-s005.docx]

**Appendix 5.** The correlation coefficient between the number of monthly hand, foot, and mouth disease (HFMD) case notifications from the Korea Disease Control and Prevention Agency (KDCA) and the monthly number of patients with HFMD from the Korea Health Insurance Review and Assessment Service (KHIRA).

| Year | 2017 | | 2018 | | 2019 | | 2020 | | 2021 | | 2022 | |
| --- | --- | --- | --- | --- | --- | --- | --- | --- | --- | --- | --- | --- |
| Months | KDCA | KHIRA | KDCA | KHIRA | KDCA | KHIRA | KDCA | KHIRA | KDCA | KHIRA | KDCA | KHIRA |
| January | 82 | 2777 | 72 | 2005 | 182 | 3864 | 110 | 2703 | 29 | 729 | 45 | 708 |
| February | 77 | 2030 | 48 | 1401 | 138 | 3035 | 106 | 1634 | 25 | 680 | 19 | 610 |
| March | 71 | 2478 | 80 | 1740 | 298 | 4705 | 35 | 1132 | 31 | 941 | 16 | 532 |
| April | 146 | 3884 | 117 | 3445 | 662 | 12827 | 41 | 1349 | 26 | 965 | 38 | 649 |
| May | 401 | 11133 | 543 | 11036 | 1825 | 40990 | 89 | 2157 | 63 | 1219 | 83 | 1387 |
| June | 1427 | 36202 | 2058 | 37336 | 7220 | 117990 | 118 | 2984 | 74 | 1716 | 185 | 4299 |
| July | 4172 | 76458 | 4435 | 82363 | 11328 | 231314 | 111 | 2636 | 77 | 2054 | 2479 | 32512 |
| August | 2018 | 45011 | 2386 | 41867 | 6267 | 103585 | 149 | 2258 | 56 | 1704 | 4033 | 100437 |
| September | 1857 | 32116 | 1733 | 26320 | 2460 | 50221 | 108 | 1468 | 103 | 1707 | 4477 | 94855 |
| October | 477 | 12303 | 701 | 13793 | 1183 | 25893 | 96 | 1216 | 134 | 1944 | 2235 | 35911 |
| November | 333 | 6730 | 546 | 9813 | 828 | 13469 | 48 | 938 | 89 | 1570 | 535 | 12512 |
| December | 148 | 3874 | 567 | 6428 | 311 | 6916 | 86 | 725 | 79 | 1084 | 295 | 4681 |
| *P-value* from Shapiro-Wilk test | 0.002 | 0.005 | 0.007 | 0.005 | 0.002 | 0.002 | 0.247 | 0.445 | 0.363 | 0.297 | 0.002 | 0.001 |
| Correlation coefficient^†^  (*P-*value) | 0.99 (*P* < 0.01) | | 0.99 (*P* < 0.01) | | 0.99 (*P* < 0.01) | | 0.81  (*P* = 0.01) | | 0.76 (*P* < 0.01) | | 0.98 (*P* < 0.01) | |

Note. KDCA: Korea Disease Control and Prevention Agency; KHIRA: Korean Health Insurance Review and Assessment

^†^Correlation coefficient: the coefficient between the number of monthly HFMD case notifications from the KDCA and the monthly number of HFMD patients from the KHIRA was obtained by Pearson’s or Spearman’s test, where appropriate.
